# The antimicrobial peptides secreted by the chromaffin cells of the adrenal medulla link the neuroendocrine and immune systems: From basic to clinical studies

**DOI:** 10.3389/fimmu.2022.977175

**Published:** 2022-08-25

**Authors:** Francesco Scavello, Naji Kharouf, Philippe Lavalle, Youssef Haikel, Francis Schneider, Marie-Hélène Metz-Boutigue

**Affiliations:** ^1^ Department of Biomaterials and Bioengineering, Institut National de la Santé et de la Recherche Médicale (INSERM), Unité Mixte de recherche (UMR) S 1121, Federation of Translational Medicine, Strasbourg University, Strasbourg, France; ^2^ IRCCS Humanitas Research Hospital, Milan, Italy; ^3^ Department of Endodontics and Conservative Dentistry, Faculty of Dental Medicine, University of Strasbourg, Strasbourg, France; ^4^ Médecine Intensive-Réanimation, Hautepierre Hospital, Hôpitaux Universitaires, Strasbourg, Federation of Translational Medicine, Faculty of Medicine, University of Strasbourg, Strasbourg, France

**Keywords:** antimicrobial peptides, chromogranins, *staphylococcus aureus*, *candida*, biomaterials, odontology, nosocomial infections, intensive care unit

## Abstract

The increasing resistance to antibiotic treatments highlights the need for the development of new antimicrobial agents. Antimicrobial peptides (AMPs) have been studied to be used in clinical settings for the treatment of infections. Endogenous AMPs represent the first line defense of the innate immune system against pathogens; they also positively interfere with infection-associated inflammation. Interestingly, AMPs influence numerous biological processes, such as the regulation of the microbiota, wound healing, the induction of adaptive immunity, the regulation of inflammation, and finally express anti-cancer and cytotoxic properties. Numerous peptides identified in chromaffin secretory granules from the adrenal medulla possess antimicrobial activity: they are released by chromaffin cells during stress situations by exocytosis *via* the activation of the hypothalamo-pituitary axis. The objective of the present review is to develop complete informations including (i) the biological characteristics of the AMPs produced after the natural processing of chromogranins A and B, proenkephalin-A and free ubiquitin, (ii) the design of innovative materials and (iii) the involvement of these AMPs in human diseases. Some peptides are elective biomarkers for critical care medicine, may play an important role in the protection of infections (alone, or in combination with others or antibiotics), in the prevention of nosocomial infections, in the regulation of intestinal mucosal dynamics and of inflammation. They could play an important role for medical implant functionalization, such as catheters, tracheal tubes or oral surgical devices, in order to prevent infections after implantation and to promote the healing of tissues.

## Introduction

In the last decades, the increasing resistance to antibiotic treatments highlights the need for the development of new antimicrobial agents (1–3). It has been estimated that drug resistant infections could annually kill 10 million people worldwide by 2050 (4). In addition to the development of multi-drug resistant bacteria (Methicillin, Vancomycin-resistant *Staphylococus aureus*), the alarming growth of fungal infections and the rise of species with intrinsic multi-drug resistance, such as *Candida auris*, are still a worldwide threat. Since the discovery of human defensins, histatins and cathelicidins, antimicrobial peptides (AMPs) have been studied, sequenced, and synthesized in laboratories with the aim of being used in clinical settings for the treatment of several bacterial, fungal and viral infections. The Antimicrobial Peptide Database (APD) contains 3324 antimicrobial peptides from six life kingdoms (391 bacteriocins/peptide antibiotics from bacteria, 5 from archaea, 8 from protists, 22 from fungi, 364 from plants, and 2446 from animals) and including some synthetic peptides (5). AMPs attack generally the phospholipid membrane *via* pore formation or other constitutive targets like peptidoglycans in Gram-negative and Gram-positive bacteria, and glucan in the fungal cell wall. Additionally, some peptides are particularly active on biofilm destabilizing the microbial communities. They can also act intracellularly on protein biosynthesis or DNA replication. Their intracellular properties are extended upon viral infection since peptides can influence several steps along the virus life cycle starting from viral receptor-cell interaction to the budding (5).

Besides representing the first defence of the innate immune system against pathogens (6), AMPs also have immunomodulatory effects working as mediators of the infection-associated inflammation, recruiting, and enhancing the activity of leukocytes and the release of cytokines, but also contributing to the control and resolution of infection (7, 8). Even if some anionic AMPs, rich in glutamic and aspartic acids, are negatively charged (9), almost all AMPs have a net positive charge for the presence of a high number of lysine, arginine and histidine (protonated in acidic conditions) (10). In addition, another common feature is represented by the hydrophobicity conferred by hydrophobic amino-acids that often overcomes 50% of the total amino acid sequence (11). The high lipophilicity, present in some part of these molecules, is useful especially for the penetration in the biological membranes. The presence of charged sequences and hydrophobic domains explains the amphipathic nature of AMPs.

The classifications are based on their structure: AMPs could be α-helix, β-sheet, linearly extended, both α-helix and β-sheet, or cyclic structures. According to their primary structure, AMPs are classified as tryptophan and arginine-rich, histidine-rich, proline-rich and glycine-rich (12). In addition to the direct antimicrobial activities, AMPs influence several other biological processes (13). They interfere with the regulation of the microbiota, wound healing, induction of adaptive immunity, and they possess anti-inflammatory, proinflammatory, anti-cancer, and cytotoxic properties, among others (13–15). Thus, due to such multifunctional nature, some authors use the broader term ‘‘host defense peptides’’ (HDPs) (13).

Response of the endocrine system to stress is characterized by the concomitant release of catecholamines from the adrenal medulla and of glucocorticoids from the adrenal cortex. Release of catecholamines, by chromaffin cells is important to maintain homeostasis during stress situation. Furthermore in addition to catecholamines, secretory vesicles of chromaffin cells release numerous proteins and peptides resulting of their endogenous processing. Also in the context of infection, the chromaffin cells release their peptides and some of these are reported as AMPs. The objective of the present review is to develop comprehensive informations for the AMPs released by the chromaffin cells of the adrenal medulla. These peptides correspond to sequences of Chromogranin A and B (CgA, CgB), proenkephalin A (PEA) and Ubiquitin (Ub). This review includes (i) the biological characteristics of these AMPs, (ii) the applications of these AMPs for the design of innovative materials and (iii) their involvement in human diseases.

## Antimicrobial peptides from the adrenal medulla

**Figure 1:**
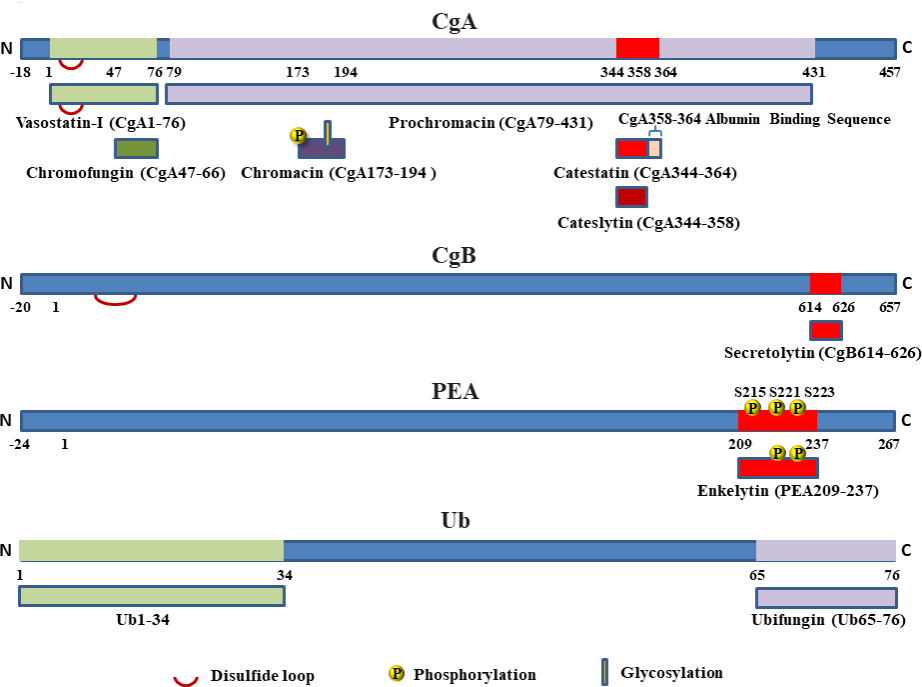
AMPs derived from the natural processing of Chromogranins Cgs (CgA and CgB), proenkephalin-A (PEA) and free ubiquitin (Ub).

A large number of biologically active peptides are synthesized as part of much larger precursor molecules (15) and the conversion of these prohormones to active peptides requires transfer from the rough endoplasmic reticulum to the Golgi apparatus, packaging into secretory granules (15), limited proteolysis by highly specific proteases and further degradation in the extracellular space after exocytotic release (15). The secretory granules of the bovine adrenal medullary chromaffin cell contain a complex mixture of secretory products, which include low-molecular constituents such as catecholamines, ascorbate, nucleotides, calcium and several water-soluble proteins. These proteins include dopamine-beta-hydroxylase, proenkephalin-A (PEA) and a family of acidic proteins (isoelectric point (pI) of 4.5-5) called chromogranins (Cgs). Cgs are secreted in the extracellular medium during stress situations by exocytosis *via* sympathetic nervous system (16). In addition, release mechanisms are dependent of environmental parameters such as pH (17). Since the first report on the circulating CgA in 1984 (18), it has become increasingly evident that Cgs are useful immunocytochemical markers for neuroendocrine tissues and are therefore frequently used as tools for the diagnosis and the follow-up of neuroendocrine tumours (19). We designed and developed a new radioimmunoassay for human chromogranin A (CgA) as diagnostic kit for cancer diagnosis and detection of metastasis. The preparation of this radioimmunoassay required large quantities of human CgA as substrate. We expressed CgA in *Escherichia coli* using a vector system and we observed that the overnight expression of CgA resulted in the complete disappearance of *E. coli*, suggesting for CgA an antibacterial activity against *E. coli*. In order to demonstrate this hypothesis, after HPLC of the proteic material present in chromaffin vesicles, we confirmed rapidly that complete CgA, several CgA-derived peptides, but also numerous peptides, included in chromaffin secretory granules and released in the extracellular medium during stress situations, possess antibacterial activity (20). These peptides, derived from the natural processing of chromogranins (Cgs) (21–26), proenkephalin-A (PEA) (27–29) and free ubiquitin (Ub) (30), are released into the circulation (blood and secretions of immune cells) [24, 26, 29] and display antibacterial (21–30), antifungal [24, 26, 30] and antiplasmodial activities (31). The AMPs derived from bovine CgA, CgB/SgI, PEA and Ub are shown in **Table 1** and **Figure 1**.

**Table 1:** Characteristics of the different AMPs derived from CgA, CgB, PEA and Ub.

Peptide	Location	Sequence	Reference
VSI	CgA1-76	LPVNSPMNKGDTEVMKCIVEVISDTLSKPSPMPVSKECFETLRGDERILSILRHQNLLKELQDLALQGAKERTHQQ	(24)
CHR	CgA47-66	RILSILRHQNLLKELQDLAL	(25)
CTS	CgA344-364	RSMRLSFRARGYGFRGPGLQL	(26)
CTL	CgA344-358	RSMRLSFRARGYGFR	(26)
CHRO	CgA173-194	YPGPQAKEDSEGP**S**QGPASREK	(23)
SEC	CgB614-626	QKIAEKFSGTRRG	(22)
ENK	PEA209-237	FAEPLPSEEEGESYSKEVPEMEKRYGGFM	(27)
UBF	Ub65-76	STLHLVLRLRGG	(30)

Phosphorylated residues are underlined and the glycosylated serine residue is in bold. The sequences correspond to bovine sequences.

Because these AMPs co-localized with catecholamines, they are released during stress for health, playing a role as a first protective barrier against bacterial infection, thus acting as factors of the innate immunity shortly after infection.

## AMPs resulting from the natural processing of proteins

### Chromogranin A

CgA is the major soluble component of the secretory granules in bovine chromaffin cells of the adrenal medulla. In mature secretory granules, about 50% of the complete protein are processed and the different fragments are released with the catecholamines (20). CgA is predominantly processed at the intragranular level, but some cleavages happen also in the extracellular medium (20). At the intragranular level, each pair of basic residues (KK, KR, RK, RR) serves as a recognition signal for endoproteolytic enzymes, such as prohormone thiol protease, PC1/3, PC2, aspartyl protease (32) and also plasmin (33). In addition, the prominent role of cathepsin L in secretory vesicles for the production of neuropeptides from their precursors has been reported (34). Furin is also involved in the processing of CgA in the chromaffin granules (35). Furthermore, secretory vesicle function involves endogenous serpin protease inhibitors for the regulation of proteolysis (36).

VS-I (**Table 1**; **Figure 1**) is a stable peptide, active against fungi and yeasts with a MIC in the range 1-10 µM (24). The short peptide Chromofungin (CgA47-66; CHR, **Table 1**) is the active domain of VS-I (25) and it is antibacterial against Gram+ bacteria (*Micrococcus luteus*) with a MIC of 0.8 µM and antifungal against fungi and yeasts with a MIC of 5-30 and 50 µM, respectively (25).

Prochromacin (**Table 1** and **Figure 1**), the C-terminal moiety of CgA (CgA79-431) was found to display antibacterial activities against *Micrococcus luteus* and *Escherichia Coli* and the complete bacterial inhibition was obtained at 1.8 µM (23). Proteolysis of prochromacin by the endoprotease Lys-C induces the formation of the antimicrobial fragment Chromacin (CgA173-194; CHRO, **Table 1** and **Figure 1**) (23). The structural analysis of CHRO indicates that its primary structure contains two post-translational modifications: a phosphorylation (Y173) and a glycosylation (S186) (**Table 1** and **Figure 1**) (23). The peptide without either phosphorylation or glycosylation is inactive, whereas the modified sequence displays antimicrobial activities against Gram+ bacteria (MIC of 1.5 µM) (23).

Catestatin (CgA344-364, CTS, **Table 1** and **Figure 1**) was identified as catecholamine release-inhibitory peptide (37) by the chromaffin cells of the adrenal medulla, but 3 years later Taylor and colleagues observed a smaller peptide, CgA344-358, with more inhibitory effects on catecholamine release (38). In addition, it was shown that CTS participates to the regulation of the secretory granule biogenesis by the disruption of the interaction CgA-phosphatidic acid interaction (39). The two peptides are positively charged, arginine-rich peptides and CgA344-358 displays antibacterial activity against Gram+ bacteria (MIC of 2 µM), Gram- (MIC of 15-50 µM), fungi (*A. fumigatus*, *F. culmorum*, *F. oxysporum*, *N. crassa*, *N. haematococca, T. mentagrophytes*) (MIC of 0.8-20 µM) and yeasts (*S. cerevisiae* and *C. albicans*, *C. tropicalis. Candida glabrata* and *C. neoformans*) (MIC of 6 -30 µM) (26). This new AMP was named Cateslytin (CTL, **Table 1** and **Figure 1**) (26). In addition, 20 µM CTS is also able to inhibit *in vitro* the growth of *Plasmodium falciparum*, (sensitive to chloroquine or not) (31).

To improve the antimicrobial activities of CTL, we prepared and tested its *D*-isomer. *D*-eniantomers resist to proteolytic degradation of microbial proteinases increasing both stability and antimicrobial activity (40, 41). The synthetic *D*-CTL isoform was tested against several microorganisms (*S. aureus*, *E. coli*, *K. pneumonia*, *Enterobacter cloacae and aerogenes, Citrobacter freundii, P. aeruginosa, C. albicans, C. tropicalis* and *C. glabrata*) showing that this modified peptide displayed a higher antimicrobial activity (40–43). We also tested the antibacterial activity of *D*-CTL against *E. coli* demonstrating that the peptide destabilizes and permeabilizes the bacterial membrane (40). The antifungal properties of *D*-CTL were also tested on *C. albicans* whether sensitive (S) or resistant (R) strains: MIC values were 5.5 µg/mL and 9.6 µg/mL respectively when the *L*-isoform is active at 50 µg/mL (42). This improved antimicrobial effect was due to a stronger stability of *D*-CTL against the proteases released by *C. albicans* (41). In addition, the *D*-isoform resists to degradation by enzymes present in saliva (41). In addition, we tested the same two isoforms of CTL on *C. tropicalis* (S and R) and *C. glabrata* (S and R) displaying for all the strains antifungal activity; finally, the highest effect of *D*-CTL was noticed at a MIC lower than 20 µg/mL (42).

The antimicrobial activities of the *L*- and *D*-isoforms of CTL were also tested against 79 highly-resistant pathogens (superbugs) (43). Specifically, *D*-CTL was active against: (i) *E. coli* (sensitive and resistant strains) between 8 µg/mL (4.3 µM) and 16 µg/mL (8.6 µM) respectively; (ii) strains of *K. pneumonia* at 32 µg/mL (17.2 µM); (iii) *E. cloacae* at 32 µg/mL (17.2 µM); (iv) *E. aerogenes* at 32 µg/mL (17.2 µM); and (v) *C. freundii* at 32 µM (17.2 µM). Furthermore, *D*-Ctl displayed antimicrobial effects against MSSA (methicilline sensitive *S. aureus*, specifically the SA112 strain) with a MIC of 32 µg/mL (17.2 µM), whereas it was less active against others MRSA strains (methiciline resistant *S. aureus*) the MIC being at 64 µg/mL (34.4 µM), against *P. aeruginosa*, the MIC varying from 64 µg/mL (34.4 µM) to 128 µg/mL (68.8 µM), and it was inactive against the highly-resistant strains L1, L2 and L3 of *C. albicans* (43).

In addition to the *D*-isomer form, several other chemical modifications were implemented in order to optimize the antimicrobial activity of CTL, such as several dimers separated but linked by different numbers of poly-ethylene glycol (PEG) spacers. Four different peptides corresponding to the dimeric form of a CTL-derived peptide with addition of a cysteine residue at C-terminal end (CTL-C) with spacers of 3, 12, 16, 46 PEG molecules were recently tested against methicillin sensitive *S. aureus* (MSSA, strain 49775), methicillin resistant *S. aureus* (MRSA, strain S1) and *C. albicans*, (ATCC 10231TM) (43). The dimer with 3 PEGs was active against MSSA, MRSA (MIC of 30 µM and 50 µM respectively) and *C. albicans* (MIC of 20 µM).

Furthermore, 18 short CTL-derived peptides including N-terminal chemical modifications such as acetylation, palmatoylation, but also C-terminal addition of tryptophan residues, were tested against several microorganisms. These modifications are unable to induce any antimicrobial activity suggesting that short peptides derived from CTL are not antimicrobial. In contrast, the *L*-CgA344-351 (R S M R L S F R) only exerted relevant antimicrobial activity against *C. albicans*, with a MIC of 50 µg/mL, indicating the crucial role of arginine residues and positive charges for the CTL-related antifungal effect (43).

### Chromogranin B/Secretogranin I

A major 13-residue peptide corresponding to the C-terminal region of CgB (CgB614-626, secretolytin, SEC, **Table 1** and **Figure 1**) has been identified into the extracellular medium from depolarized cultured chromaffin cells (21). A strong relationship between the sequence of this new antibacterial peptide named SEC and cecropins was reported (21). Cecropins are a family of antimicrobial peptides that were first isolated from the hemolymph of *Drosophila* (44), then, from the giant silk moth *Hyalophora cecropia*, and subsequently from numerous insects (45). The cecropin family is characterized not only by antimicrobial and antifungal properties, but it also has anticancer properties (46). The antitumor potential is confirmed by *in vitro* studies conducted on several different cell lines, among others, prostate and breast cancer cell lines (46). SEC was found to display antibacterial activities against *Micrococcus luteus* (21). We observed the natural formation of a pyrolidone glutamic acid at the N-terminal end. SEC and its pyrolidone glutamic derivative display antibacterial activities reaching 100% inhibition at 2 µM. SEC is also active against *Bacillus megaterium* (around 80% of inhibition for 2 µM). We designed different peptides to test the antibacterial activities. We pointed out the importance of: (i) the length of the peptide to interact with the bacterial membrane; (ii) the presence of basic residues along the sequence (K2, K6, R11); (iii) the presence of hydrophobic residues I3 and F7 (21).

The structural properties of SEC were assessed by two-dimensional NMR techniques (NOESY, two-dimensional nuclear Overhauser effect spectroscopy and HOHAHA, homonuclear Hartmann-Hahn spectroscopy). In aqueous solution, secretolytin presents a conformational flexibility; the structural homology of SEC with cecropins suggests that the alpha-helical amphipathic domain may underlie its antibacterial activity (22).

### Proenkephalin-A

We have also investigated the presence of AMPs derived from PEA. Soluble proteins from chromaffin granules were separated using three successive HPLCs (27). After the last HPLC, using Edman sequencing, we characterized three active and one inactive antimicrobial fractions. The four fractions contain the fragment with its N-terminal sequence located at position PEA209-237. The structural differences were identified by use of matrix assisted laser desorption ionization time-of-flight mass spectrometry (MALDI-TOF). The antibacterial bisphosphorylated form of this sequence was identified in addition to the inactive monophosphorylated, triphosphorylated and non-phosphorylated peptide (27). The three phosphorylated serine residues had been previously identified in position S215, S221 and S223 (47). For the antibacterial form of the sequence PEA209-237 the two phosphorylated sites were identified in position 221 and 223 by use of the ethanethiol method (48). The antibacterial bisphosphorylated peptide PEA209-237 was named Enkelytin (ENK, **Table 1** and **Figure 1**). It inhibits the growth of *Micrococcus luteus* and *Bacillus megaterium* with a MIC evaluated at 200 nM, but it was inactive against *E. coli*. To demonstrate that the antibacterial activity was not due to non-peptidic material, we submitted the active material to a tryptic digestion over 18h at 37°C. The resulting mixture of tryptic peptides was completely inactive against *M. luteus* growth.

Sequence alignment of ENK with Diazepam Binding Inhibitor (DBI) (49) was performed by use of Clustal V and we observed the alignment of 10 hydrophilic residues and 4 hydrophobic residues (28). The ENK model was built taking into account the similarity of sequences between Enkelytin and DBI and using the program Pro-Explore (Oxford Molecular). In the ENK sequence, the two phosphorylated serine residues S221 and S223 are clustered near acidic residues that may induce the opening of the boomerang angle and thus help to the lytic activity (27). Then, an extensive study has been carried out on ENK and the presence of helical structure was confirmed in the ^1^H NMR spectra (28). ENK sequence contains three proline residues which are able to adopt either the *cis* or the *trans* conformation of the peptide bond. The three-dimensional structure of ENK adopts a *L*-shape and the negatively charged phosphate groups probably induce conformational change by electrostatic interactions with the glutamic residues (S221/E228 and S223/E230) (28).

In order to characterize the biological function of ENK, we have examined whether it is released from chromaffin cells, and whether it could be detected in infectious fluids and in polymorphonuclear secretions following stimulation. After HPLC of these biological fluids, ENK was characterized from secretions of chromaffin cells and from cow knee periarthritis abscess fluid (29).

To conclude the antibacterial ENK may originate from chromaffin and immune cells, such as polymorphonuclear cells (PMNs) and macrophages (50). In addition, PEA the precursor of ENK is synthesized and processed within various types of immune cells at the site of inflammation (51), suggesting a primordial role in the connection between neuroendocrine and immune systems.

### Ubiquitin

Ubiquitin (Ub) is a peptide of 76 residues, found in all eukariotic cells and with a well-conserved sequence from protozoans to vertebrates (52–54). It plays a major role in the process for selective protein degradation.

By using Western blot analysis, we reported the subcellular localization of free Ub in bovine chromaffin cells. Ub is present in secretory granules and secreted with catecholamines upon chromaffin cells stimulation. After HPLC of the secretions of stimulated chromaffin cells, Ub was identified by use of Edman sequencing and MALDI-TOF analysis. The antimicrobial assays show that: (i), Ub displays antimicrobial activity against *M. luteus*, *B. megaterium* and *N. crassa* at a MIC of 60 µM. In order to characterize the shortest active peptide derived from Ub we submitted Ub to a digestion with the endoproteinase Glu-C and HPLC. We focused on the active fraction and, after sequencing the fragment, Ub65-76 (ubifungin, UBF, **Table 1**, **Figure 1**) was identified. It is active against *M. luteus* and *B. megaterium* with a MIC of 10 µM. In contrast, no activity is detectable for concentrations up to 100 µM against Gram negative bacteria (30). UBF is also active against filamentous fungi such as *A. fumigatus*, *N. haematococca*, *F. culmorum*, *F. oxysporum*, *T. mentagrophytes* and *T. viride* and yeast forms such as *C. albicans, C. tropicalis, C. neoformans* and *C. glabrata* with a MIC 15-20 µM. Because we could not obtain a restart of the microorganisms after 48h of treatment with UBF, we propose that the antimicrobial activities were due at a lytic effect. In addition, UBF is not toxic for erythrocytes at a concentration up to 50 µM (30).

The three dimensional structure of Ub reveals that the residue E64 is located close to the N-terminal sequence surrounding F4 (55). A weak antifungal activity was reported for 100 µM Ub1-34 against *N. crassa*, but when 10 µM Ub1-34 was added to UBF, the two peptides were found to act synergistically to inhibit the growth of filamentous fungi and yeasts (*N. crassa, A. fumigatus, T. mentagrophytes, T viride, C. albicans, C. tropicalis, C. glabrata* and *C. neoformans*). The confocal laser microscopy indicated that 1 µM rhodaminated UBF was visible at the level of the bacterial cell wall and in the inner compartment after 2 min of incubation. In addition, confocal laser microscopy and antimicrobial assays confirm that the N-terminal fragment Ub1-34 potentializes the effects of UBF (30).

## Interaction of the AMPs with calmodulin

The AMPs present in chromaffin cells act by destabilizing the cell wall, but we have investigated whether they could interact with intracellular targets. Previously, it had been reported that CgA and Ub interact *in vitro* with Calmodulin (CaM) with the formation of covalent linkages (56, 57). It was also reported that Calcineurin (CaN), the Calmodulin-activated phosphatase B plays an important role in the hyphal growth of filamentous fungi (58). Thus, the possibility for CHR and UBF to inhibit the phosphatase activity of CaN was tested: CHR and UBF at 50 µM inhibit 85% of the phosphatase activity (25, 30).

## Potentialization effect of CHR and UBF

When CHR and UBF were tested together against filamentous fungi (*N. crassa*, *T. mentagrophites*, *A. fumigatus*) and yeast (*Candida*) a synergistic effect was obtained (59). For *N. crassa*, *T. mentagrophites*, *A. fumigatus* and *Candida* the MICs of UBF, CHR and UBF+CHR were (10, 5, 2 + 2 µM), (20, 16, 7 + 5 µM), (30, 16, 10 + 5 µM) and (16, 10, 5 + 5 µM) respectively (59).

## Synergistic effects of CTL with antibiotics

Because AMPs interact with membranes, they represent helpful candidates to potentiate antibiotics through synergistic interactions. Antimicrobial tests were carried out using CTS, amidated CTS and therapeutic antimicrobial drugs (minocycline and voriconazole). Combinations of *D*-Ctl with classical antibiotics, such as Cefotaxime, Amoxicillin, Methicillin and Voriconazole show a synergistic effect (40, 41). The Fractional Inhibitory Concentration (FIC) were evaluated and FIC>0.5 corresponds to a synergistic effect, 0.5<FIC<2 corresponds to an additive effect and FIC>2 corresponds to an antagonist effect (60). The combination of amidated CTL and minocyclin led to a FIC of 0.37 against *S. aureus*. For CTL and voriconazole a FIC of 0.25 and 0.5 against *C. albicans* and *C. tropicalis* were respectively obtained.

It would reasonably be proposed that mechanisms exist by which the AMPs (one or combinations) could favor the destabilization of the membrane of the pathogen allowing a rapid penetration of the antibiotic inside the microorganisms and more efficiently reach its site of action.

## Synergy of Cts-derived peptides with antibiotics and albumin

Recently, we have demonstrated that CTS, but not CTL, interacts with circulating albumin suggesting the important role of the C terminal part of CTS for the binding (**Figure 1**). The interaction CTS/albumin improves the antimicrobial activity of the peptide against *C. albicans* at a concentration of 4 µM, demonstrating a synergistic effect (42).

## Effects of bacterial supernatants against AMPs released from the adrenal medulla

*A S. aureus* supernatant was tested against the proteic material released by chromaffin cells (61). After HPLC and antimicrobial assays, we showed that the major antibacterial peptides derived from CgA after processing (CHR, CTS) were degraded and that the resulting fragments displayed antifungal activities. Interestingly, CTL, the active domain of CTS resists to *S. aureus* and *Salmonella enteritidis* supernatants.

## Antimicrobial biomaterials including CgA-derived peptides

The contamination of surgical sites and the subsequent nosocomial infections are often related to microbial biofilms development on biomaterials (62, 63). It was found that different AMPs prevent the formation of a biofilm on materials (64, 65). Currently, different biomaterials functionalized by peptides and peptidomimetics agents are available for clinical applications including oral and wound sites, but also for medical devices implanted in different organs. Several biomaterials including AMPs are designed to combat biofilm-associated infections with functionalized anti-adhesive surface coatings or self-killing system with controlled release of AMPs (66). Several studies demonstrate that the more active antimicrobial CgA-derived peptides are elective candidates for medical-implant functionalization, such as catheters, tracheal tubes or oral surgical devices, in an attempt to prevent fungal or bacterial infections after implantation. In 2005, we designed an antifungal coating for biomaterials by the layer-by-layer technique and functionalized it by the insertion of CHR (**Table 1**). In this context, the peptide preserved its antifungal activity against *C. albicans* and *N. crassa*, CHR being able to penetrate into microbial membranes and kill fungi (67). This first biomedical application of antimicrobial CgA-derived peptides was for oral medical devices; its biocompatibility was assed demonstrating that human gingival fibroblasts proliferated on this biomaterial but it also displayed its antifungal and non-toxic effect *in vivo* in an oral candidiasis rat model (67). Furthermore, other CgA-derived peptides were used to prevent oral cavity infections in hydrogels coating (68). Thus, CTL was used to functionalize an alginate hydrogel with catechol moieties or with thiol-terminated pluronic components. These two typologies of gels were used to strongly improve the adhesive and cohesive properties of the gel on surface gingiva. Moreover, the embedded CTL in the two gels display a strong antimicrobial activity against *Porphyromonas gingivalis* without toxicity on human gingival fibroblasts (68).

In addition, CTL was also included in biocompatible and biodegradable polysaccharide multilayer films of hyaluronic acid in order to functionalize biomaterial coating with antibacterial and antifungal properties (69). Furthermore, the same multilayer films were also functionalized with CTL-C and hyaluronic acid as polyanion and chitosan as polycation (HA-Ctl-C/CHI). After a 24h- incubation with *S. aureus* or *M. luteus* or *C. albicans*, the biofilms fully inhibited the development of the aforementioned pathogens (69). This innovative approach takes advantage over the secreted hyaluronidase by pathogens, leading to biofilm degradation and a consequent release of CTL. Of great interest for medical applications, these biomaterials limited fibroblasts adhesion, showing no cytotoxic effects on these cells (69).

Along with infective issues, biomedical device implantations are often problematic for the adverse immune reaction related to allergenic material components, such as metallic compounds (70). For this particular issue, CgA-derived peptides were ideal molecules to functionalize biomaterials. Of note, a silver platform coated with a polyelectrolyte multilayer composed of polyarginine and hyaluronic acid was functionalized with CTS (71). It provided antimicrobial effects against *S. aureus*, *C. albicans* and *A. fumigatus* and, more interesting, it strongly inhibited the production of inflammatory cytokines in human primary macrophage contributing to a reduced inflammatory reaction when compared with classical silver implants. This system may allow the correct integration of silver devices reducing potential inflammatory response and microbial infections after implantation (71).

### DOPA_5_T-CTL

CTL was also used to design a synthetic system for on-demand *S. aureus* self-killing (43). A tri-blocks peptide, called DOPA_5_T-CTL, was composed by association of a DOPA_5_ (dopamine_5_) group (DOPA-K-DOPA-K-DOPA), a sequence TLRGGE recognized and cleavable by the endoprotease Glu-C specifically produced by *S. aureus* (72) and, finally, a CTL sequence. The dopamine system is inspired by the adhesive substance of sea mussel (73). It is an adhesive material that allows to coat almost any surface while having many advantages: easy to synthesize, easy to functionalize and it is biocompatible (73). In recent years, this system has been described for the coating of several synthesized nanoparticles and to functionalize biomaterials by the rapid film formation and the non-specific coating with all materials (74).

The dopamine DOPA_5_T-CTL was active against MSSA (strains 25923, 49775) and MRSA (strain S1 and V8) at MIC of 20 µM, 45 µM, and 65 µM respectively. In addition, the DOPA_5_ sequence facilitated the release of the full peptide CTL after action of the Glu-C endoprotease in comparison with T-CTL, which is degraded (43).

**Figure 2:**
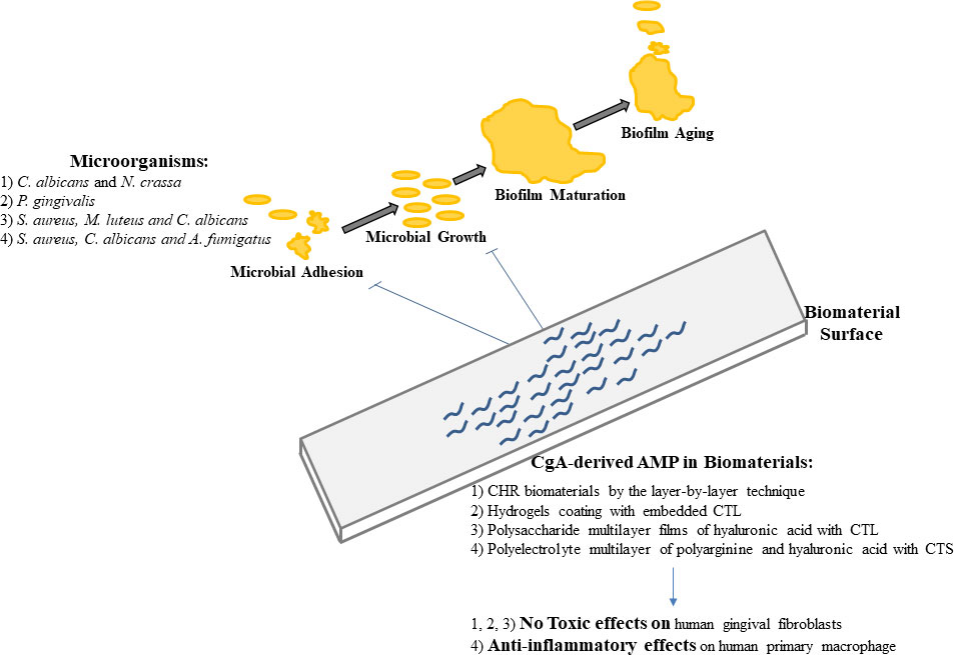
Pathogens biofilms development and roles of CgA-derived AMPs for the prevention of biomaterial contaminations.

All these data demonstrate that CgA-derived peptides preserve their antimicrobial activity and may produce potential biomedical applications when included in biomaterials (**Figure 2**). The very active antimicrobial CgA-derived peptides are elective candidates for medical implant functionalization such as catheters, tracheal tubes or oral surgical devices, in order to prevent care-related infections by fungi or bacteria.

### CgA-derived peptides and cell culture

CgA-derived peptides are also proposed as alternative to antibiotics in an innovative system for cell cultures. An extracellular matrix mimicking film was designed to allow the cellular growth without adding serum, growth factors and antimicrobial agents (75). Concerning this last point, functionalization with AMPs was performed with CTS and *S. aureus* to assess the antibacterial properties of the gelatin film and comparing CTS effects to antibiotics mixture. After one day of incubation, bacteria growth was strongly inhibited in CTS embedded film with similar effect of antibiotics film counterpart (75).

## The involvement of the AMPS released from the adrenal medulla in human diseases

### CgA and VS-I with patients in Intensive Care Unit

Patients admitted to hospital or requiring chronic device implantations pay a high toll to infection related to care (76). In critical care departments, where the incidence and the severity of such nosocomial infections is even higher than elsewhere, the problem is even more serious because antimicrobial resistance poses a major threat to human health (76). Thus, septic shock which is the most severe form of infection is a major risk for critically ill patients because they are frequently weakened by failing immune defense whatever reasons (77). Infection and pathogen invasion is associated to adrenergic stimulation as a part of the fight and fly reaction to a health stress, and consequently, an increased secretion and processing of CgA derived-peptides occurs (78, 79). It was previously reported the role of this protein as biomarker for mortality in Intensive Care Unit (ICU) patients with sepsis showing that higher CgA plasma levels were associated to mortality (80–82). In the present review we focus on the expression of AMPs released from the adrenal medulla during human diseases, surgery and dental infections.

To understand the role of VS-I in the context of septic shock, we evaluated plasmatic levels of VS-I in a study of critically ill patients with sepsis and in 30 healthy subjects. Plasma levels of VS-I were found gradually increased in patients with sepsis, severe sepsis and septic shock in comparison to healthy controls, also indicating in this setting that plasmatic VS-I is more sensitive and specific for the evaluation of sepsis and its severity than CgA measurement (83). In 2012, the plasmatic concentrations of VS-I were assessed in 481 non-selected critically ill patients in a multicenter study, in order to determine its role as a biomarker of severity without taking into account any specific primary diagnosis (84). Furthermore, the level of this peptide was also compared to classical biological markers of acute damage in critical care medicine and mortality was monitored after 28 days. Critically ill patients showed significantly increased VS-I levels at hospital admission in comparison with healthy controls and, more interesting, survivor patients displayed relevant lower plasmatic VS-I than non-survivors (84). Indeed, patients with circulating VS-I concentrations beyond a value of 3.97 ng/mL (0.46 nM) had better survival rate (84). Finally, we reported that admission value of VS-I was crucial independent parameter for predicting mortality in critically ill patients when associated with lactate and age (84).

Of note, *in vitro* studies reported the interaction of VS-I with classical circulating proteins such as albumin. These interactions resulted in relevant biochemical associations through disulfide loop (S17-S38). (85). This interaction and the antioxidative properties of albumin allowed restoration of the oxidative status essential for the antimicrobial activity of VS-I (85). In a randomized clinical trial involving ICU patients with circulatory failure and with serum albumin lower than 20 g/L, the continuous infusion of 4% albumin was associated with a significant decrease in both site colonization (4 *vs* 12 episodes; p = 0.035), and nosocomial infections number (2 *vs* 13 episodes; p = 0.002) when compared to controls infused with hyperoncotic albumin. This study confirms *in vivo* that 4% therapeutic albumin can increase VS-I availability for better antimicrobial defense.

**Figure 3:**
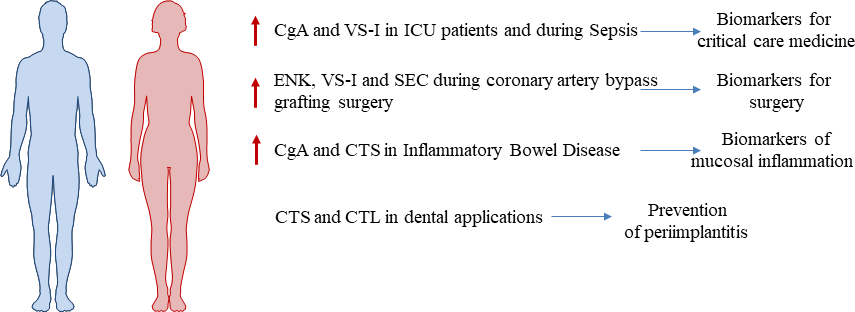
AMPs released from the adrenal medulla as biomarkers of human diseases.

In conclusion, these data support the concept that circulating CgA could be used as specific prognostic biomarker of severity in the absence of other causes for increased circulating CgA. VS-I may even better indicate prognosis if combined with age and lactate for triage in the emergency department (**Figure 3**).

### ENK, VS-I and SEC during coronary artery bypass grafting surgery

The AMPs released by chromaffin vesicles represent a potential innate defense mechanism against microbial infections. However, whether this process occurs in humans undergoing surgical interventions, is only poorly documented. An increase in the release of AMPs during coronary artery bypass (CBP) grafting was reported: ENK was initially present at low level in plasma and was then released in increased amounts as early as just after skin incision (86) (**Figure 3**). It was also demonstrated that ENK was metabolized *in vivo* to Met-enkephalin peptides that have granulocyte chemotactic activity. Peptides VS-I and SEC were also released in increased amounts just after skin incision (87). A combination of techniques including confocal microscopic analysis, mass spectrometry measurement, and antibacterial tests allowed for the identification of the positive role of interleukin 6 (IL-6) in the SEC release from monocytes *in vitro*. Because IL-6 release is known to be strongly enhanced during CPB, we suggest a possible relationship between IL-6 and the increased level of SEC in patients undergoing CPB.

### CgA and CTS in inflammatory bowel disease

In inflammatory bowel diseases (IBD), the mucosal inflammation is characterized by an alteration of CgA production. It was reported an increase for CgA and CTS (88) (**Figure 3**). In an experimental mouse model of IBD, a treatment with CTS suppresses exacerbated inflammatory responses and decreases intestinal inflammation through the modulation of the M1 macrophage and the down regulation of pro-inflammatory cytokines (89). It was also reported that CTS modulates gut microbiota composition under non-physiological conditions (90). Complex and diversified microbial populations colonize the mammalian intestinal tract. CTS-treated mice had a lower relative abundance of *Firmicutes* and higher abundance of *Bacteroidetes*. On biopsies harvested from patients with active ulcerative colitis, it was also reported that CST regulates intestinal injuries and dynamics (91).

### CTS in dental applications

Dental plaques are covered by a large variety of bacteria which are the major reason for dental caries occurrence; *Streptococci spp* are the predominantly strains present. These bacteria, due to the production of acids, can demineralize and affect tooth tissues (92). Different materials, solutions and additives are used in dental treatments in order to decrease and eliminate the amount of bacteria in oral cavity and teeth tissues. Calcium silicate based materials were used in dental treatment due to its bioactive reactions as antibacterial activity and remineralization effects (93). Other materials and solutions such as sodium hypochlorite and chlorhexidine were used in root canal treatment to disinfect and eliminate resistant *bacteria* in root canal system and dentinal tubules (94). Moreover, natural molecules are used in oral environment such as polyphenols, which have biological benefits as they are natural antibacterial and antioxidant molecules (95). These different materials are made commercially available in dental market and used worldwide even though they sometimes display cytotoxicity (96).

Along the last two decades, attention has been paid to the use of AMPs (97). Synthetic AMPs were developed in order to be used as antimicrobial agents against several oral diseases. The *L*-CTS combined with bovine serum albumin and *D*-CTL display an important role as antifungal agents against oral candidosis (42).

These new antimicrobial agents were used in different dental fields such as (i) endodontic treatment, (ii) caries managing and (iii) the modification of dental materials in order to ameliorate the biological effects of these materials in oral cavity.

An original hydrogel, Alginate–Catechol (Alg–Cat), combined to *D*-CTL was designed to prevent periimplantitis induced by *P. gingivalis* that grow in dental implants surrounding tissues and between implants and abutments (98). Gelation was fast and achieved in 2 min with a storage modulus between 25 and 30 Pa. The gels were stable under strain and showed an adhesive potential reinforced with aging at 18 h (5.4 kPa) under a slow retraction speed (4 J·m^−2^ at 10 µm/s) with a mixed rupture profile (adhesive/cohesive). The MIC of *D*-CTL inside the Alg–Cat gel against *P. gingivalis* was equal to 470 µg·mL^−1^ after 24 h. Peptides recovered in the supernatant and inside the gel were fragmented, most of them conserving the α-helix active site. No bacteria were visualized at the surface and inside the gel after 24 h. This gel is promising for clinical application for the prevention of periimplantitis.

Finally, the AMPs reported in this review may play an important role in the protection of oral infections, regulate the inflammation and they could thereby play an important role with dental materials in order to insure antimicrobial and antifungal activities and remineralization process in improving healing of oral tissues (**Figure 3**).

## Conclusions

The unexpected discovery of these AMPs has allowed to establish a strong link between neuroendocrine and immune systems. The three families of peptides correspond to molecules with important roles in fundamental functions of mammals. Cgs and its derived-peptides correspond to stress hormones and are largely reported as belonging to a protective system for the maintenance of integrity and the regulation of inflammation. PEA and its derived peptides participate to the release of enkephalins in relation with the regulation of pain, but are also involved in immune regulations. The biological roles of Ub are crucial with the complex degradation process of unfolded proteins and with the control of the inflammasome.

Finally, these AMPs (alone, or in combination with others and antibiotics) might play an important role in the innate defense with the protection against infections, the prevention of nosocomial infections and the regulation of inflammation. Interestingly, CTL the active domain of CTS resists to *Staphylococcus aureus* and *Salmonella enteritidis* supernatants. In addition, to the direct antimicrobial activities and the regulation of the adaptive immunity, some peptides display others fundamental functions, such as the regulation of intestinal mucosal dynamics and the regulation of microbiota.

In relation with multi-resistance of pathogens to antibiotics, an alarming issue in human medicine (76), one could imagine a mechanism by which the AMPs (alone or in combinations) could favor the destabilization of the membrane of pathogens, allowing the rapid penetration of the antibiotic inside microorganisms with more efficiency in reaching its site of action.

The more active antimicrobial CgA-derived peptides are elective candidates for medical implant functionalization in order to prevent fungal or bacterial infections after implantation. Their use with biomaterials to insure antimicrobial and antifungal activities is stimulating but requires supplementary experiments until daily practice. In the future, the design of low cost molecules mimicking the active peptides may constitute new objectives.

## Author contributions

Conceptualization: FS, M-HM-B, PL, YH. Preparation of original draft: M-HM-B, FS, FSch, NK. Review and editing: FS, FSch. All authors contributed to the article and approved the submitted version.

## Funding

All the authors were supported by Inserm, the Faculty of Odontology, the University of Strasbourg, the Hôpital universitaire de Strasbourg. Francesco Scavello was supported by the University ‘Italo-Francese’ in the context of ‘Vinci Project 2014’ (n° C2-72) attributed to the Department of Biology, Ecology and Earth Science University of Calabria, Arcavacata di Rende, Italy and the INSERM_UMR 1121, Biomaterials and BioEngeneering, Strasbourg, France.

## Acknowledgments

The authors thank Inserm, the Faculty of Odontology, the University of Strasbourg, the Hôpital universitaire de Strasbourg for their financial support, Dr Dominique Aunis for his full support at the starting of these researches. Prs Karen Helle, Angelo Corti, Jean-Eric Ghia for the stimulating discussions, Dr Gille Prévost for the studies with immune cells, Drs Jean-Marc Strub, Karine Kugardon, Yannick Goumon, Jenny Briolat, Anne-Estelle Kieffer, Rizwan Aslam, Menonve Atindehou, Abdo Zaet, Pauline Dartevelle, Francesco Scavello for the work realized during their thesis, Mrs Sophie Hellé and Cosette Betscha for the excellent technical assistance, Pr Corti and CisBio for the generous gift of CgA and VS-I ELISA assays.

## Conflict of interest

The authors declare that the research was conducted in the absence of any commercial or financial relationships that could be a potential conflict of interest.

## Publisher’s note

All claims expressed in this article are solely those of the authors and do not necessarily represent those of their affiliated organizations, or those of the publisher, the editors and the reviewers. Any product that may be evaluated in this article, or claim that may be made by its manufacturer, is not guaranteed or endorsed by the publisher.
